# Bioelectrical Signals and Ion Channels in the Modeling of Multicellular Patterns and Cancer Biophysics

**DOI:** 10.1038/srep20403

**Published:** 2016-02-04

**Authors:** Javier Cervera, Antonio Alcaraz, Salvador Mafe

**Affiliations:** 1 Dept. de Termodinàmica, Facultat de Física, Universitat de València, E-46100 Burjassot, Spain; 2 Dept. de Física, Laboratori de Biofísica Molecular, Universitat “Jaume I”, E-12080 Castelló, Spain

## Abstract

Bioelectrical signals and ion channels are central to spatial patterns in cell ensembles, a problem of fundamental interest in positional information and cancer processes. We propose a model for electrically connected cells based on simple biological concepts: *i*) the membrane potential of a single cell characterizes its electrical state; *ii*) the long-range electrical coupling of the multicellular ensemble is realized by a network of gap junction channels between neighboring cells; and *iii*) the spatial distribution of an external biochemical agent can modify the conductances of the ion channels in a cell membrane and the multicellular electrical state. We focus on electrical effects in small multicellular ensembles, ignoring slow diffusional processes. The spatio-temporal patterns obtained for the local map of cell electric potentials illustrate the normalization of regions with abnormal cell electrical states. The effects of intercellular coupling and blocking of specific channels on the electrical patterns are described. These patterns can regulate the electrically-induced redistribution of charged nanoparticles over small regions of a model tissue. The inclusion of bioelectrical signals provides new insights for the modeling of cancer biophysics because collective multicellular states show electrical coupling mechanisms that are not readily deduced from biochemical descriptions at the individual cell level.

The modeling of spatial patterns in multicellular ensembles is relevant to positional information processes (e.g. embryogenesis) and cancer initiation and progress^1^,^2^,^3^. Much progress has been achieved on the basis of genetic concepts and specific biochemical signals. Recently, different studies have noted a number of difficulties with existing models, encouraging non-specific, biophysically-oriented approaches which may add new ideas to the field^4^,^5^,^6^,^7^,^8^. Bioelectrical signals should be included in these approaches because ionic species and electrical phenomena are crucial for cell function. Indeed, cells have a significant membrane potential of the order of tens of millivolts, the cell inside being at a negative electrical potential with respect to the extracellular medium^2^,^6^,^9^. This potential can be involved in the cell cycle and is regulated by the protein ion channels in the cell membrane (in particular, by the voltage-gated inward and outward-rectifying channels^9^,^10^,^11^).

We propose a highly-idealized approach for an electrical network of non-neural model cells where the biochemical coupling of the multicellular ensemble with the external microenvironment is realized by an ion channel blocker. The approach is based on a reduced number of biological assumptions: *i*) the membrane potential value of a single cell, assumed to be regulated by the concerted action of hyperpolarizing (inward rectifying) and depolarizing (outward rectifying) voltage-gated ion channels^9^,^12^, characterizes the cell electrical state (polarization); *ii*) the long-range electrical coupling of the cells is realized by a network of gap junction channels between neighboring cells^13^,^14^; and *iii*) the spatial distribution of a biochemical agent in the external microenvironment can modify locally the conductance of the ion channels (and then the cell membrane potential)^9^,^15^. The spatial patterns emerging from this model are characterized by the map of cell potentials, which are ultimately regulated by the voltage-gated channels because of their externally tunable electrical conductance^9^,^10^,^11^,^12^,^16^,^17^. We explore the consequences of this bioelectrical map on spatial patterning, the role of ion channels in the modeling of cancer biophysics, and the distribution of charged nanoparticles over multicellular ensembles. We concentrate on the electrical effects which may occur in small multicellular regions, ignoring the slow diffusional processes (e.g. changes in the ionic concentrations) which should follow the relatively fast electric responses.

This biophysical approach constitutes a significant extension of a previous model^14^ which is applied here to new problems of current interest. The model is an oversimplification of real biological systems but it incorporates an experimental fact which has traditionally been disregarded: the coupling between cells involves not only biochemical but also bioelectrical signals^2^,^11^,^18^,^19^,^20^. Indeed, morphogenetic fields acting on the tissue morphology (embryo), morphostatic fields keeping the tissue microarchitecture (adult), and cancer resulting from the disruption of this architecture have usually been studied on the basis of biochemical concepts at the single cell level^1^,^21^. In particular, cell genetics and diffusion-reaction processes are the basic approaches for the modeling of spatial patterns in multicellular ensembles. While these dominant approaches have shown the crucial role of genetics and biochemical signals, recent studies have also noted the importance of bioelectrical signals and communication at the multicellular level^2^,^4^,^8^,^14^,^22^,^23^,^24^, especially for the modeling of cancer initiation and progress^6^,^11^,^22^,^25^,^26^.

Cancer cells tend to be depolarized (low membrane potential in absolute value), which has been connected with overexpression of specific ion channels^27^. In particular, upregulation of sodium channels and downregulation of potassium channels has been ascribed to carcinoma cell lines and tissues^7^. The blocking of specific channels by an external agent could reverse the membrane depolarization, acting to reduce the oncogenic process. In addition, depolarization of the cell membrane caused by channel overexpression produces significant changes in the spatial distribution of negatively charged lipids and their interactions with positively charged proteins^27^. These changes may activate biochemical pathways which promote uncontrolled cell proliferation (see Fig. 1 of ref. 27). The above experimental facts suggest that changes in specific ion channels regulating the single cell membrane potential can promote long-range processes because of the biochemical and bioelectrical couplings underlying multicellular organization^2^,^13^,^20^. In an effort to illustrate the consequences of the bioelectrical coupling, our model simulations explore the cases of ion channel upregulation and blocking, defective multicellular communication, and spatial patterning. The conclusions obtained are related with current experimental approaches and suggest that bioelectrical signals and ion channels should be of particular significance for the modeling of cancer biophysics.^6^,^7^,^10^,^11^

## Biophysical Model

### Description of the cell electrical state

**Figure 1:**
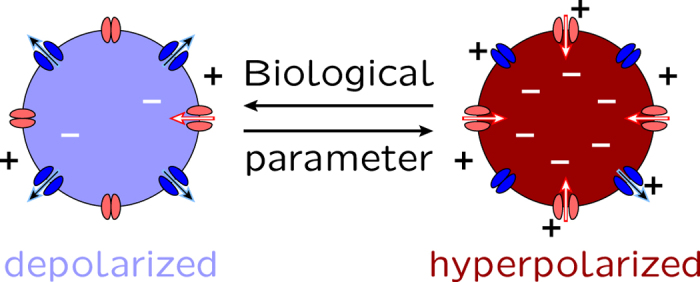
The model cell as a dynamical system undergoing transitions between the low (depolarized, abnormal) and high (hyperpolarized, normal) *V*_m_ states. In this model, the transitions are associated with changes in the biological parameters which determine the electrical rectification characteristics of the voltage-gated channels. The ion channels in the cell membrane are schematically shown. We consider that the inward-rectifying channel (inward arrow) favors the hyperpolarization while the outward-rectifying channel (outward arrow) favors the depolarization.

The cell electrical state is described by the membrane potential *V*_m_ < 0, which is defined as the potential difference between the cell cytoplasm and the extracellular microenvironment under zero current conditions. This potential difference regulates the entry of sodium, potassium, calcium, and biologically-relevant molecules to the cell^7^,^9^,^10^. Figure 1 schematically shows the model cell as a dynamical system undergoing transitions between the low (depolarized, abnormal) and high (hyperpolarized, normal) values of *V*_m_^12^. These transitions may be associated with changes in biological parameters such as the pH and the ionic concentrations of the salt solutions^9^,^25^ but we concentrate here on the electrical characteristics of two model voltage-gated channels. These channels are involved in membrane hyperpolarization/depolarization processes and cancer, as shown in experimental studies^6^,^10^,^11^,^28^,^29^,^30^,^31^. Indeed, the negative membrane potential value is a physiologically-relevant cell characteristic: anomalous inward-rectifying potassium channels found in tumor cell lines give values of *V*_m_ different than those found in normal cells^6^,^25^ and changes in the regulation of voltage-gated sodium channels are associated with depolarized values of *V*_m_ and cancer^7^,^28^,^29^. Note however that different ion channels and pumps, in addition to potassium and sodium ions, are implicated in cancer (see e.g. Table 1 of ref. 6 and references therein). Abnormally-low absolute values of *V*_m_ correspond to plastic cells while high absolute values of *V*_m_ are found in terminally differentiated cells^25^,^32^.

**Figure 2:**
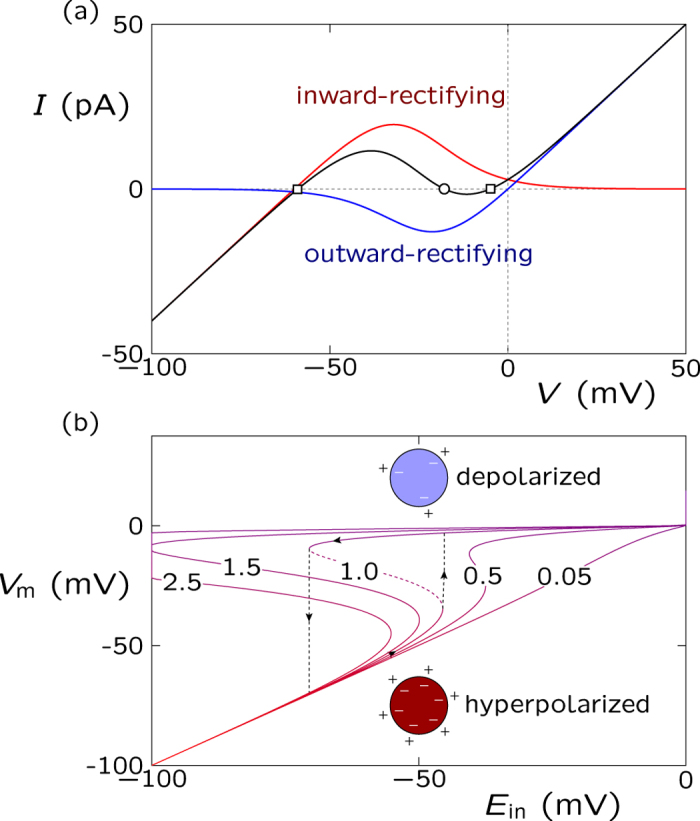
The ion channel current-voltage curves and the membrane potential. (**a**) The outward and inward-rectifier currents (*I*_out_ and *I*_in_) and the total current (*I*_in_ + *I*_out_) (central curve) as a function of the voltage *V* for two model channels of maximum conductances *G*_in_ = *G*_out_ = 1 nS, threshold potentials *V*_th,out_ = *V*_th,in_ = −25 mV, and equilibrium potentials *E*_in_ = −60 mV and *E*_out_ = 0 mV. The two stable (outer squares) and the unstable (central circle) membrane potentials shown in the total current curve are obtained from the condition of zero current, *V*_m_* = V*(*I* = 0). The stable values correspond to the hyperpolarized (−60 mV) and depolarized (0 mV) potentials, approximately. In this model, the inward-rectifying channel acts to fix the hyperpolarized membrane potential while the outward-rectifying channel favors the depolarized value (see Fig. 1). (**b)** The membrane potential *V*_m_ as a function of the equilibrium potential *E*_in_ parametrically in the conductance ratio in *G*_out_/*G*_in_ (the numbers on the curves). The curve corresponding to *G*_out_/*G*_in_ = 1.0 shows the transitions (arrows) between the depolarized and hyperpolarized stable potentials while the dashed line corresponds to the unstable potential branch^14^.

While there is a multiplicity of ion channels which make contributions to *V*_m_^9^,^11^, the basic characteristics of the membrane potential bi-stability shown in Fig. 1 are illustrated using the physiologically significant outward and inward-rectifying channels in Fig. 2a^12^,^14^. These channels can mimic the bioelectrical properties typical of sodium^10^,^11^ and potassium^10^,^11^,^30^,^31^ channels and are introduced here as a simple model for membrane potential regulation. Note that we ignore the effects of membrane ion pumps in Fig. 2a, assuming that passive ion transport determines the electrical potential across the plasma membrane of animal cells to a large extent^13^.

The current (*I*)-voltage (*V*) curve in Fig. 2a is determined by adding the contributions of the inward and outward-rectifying channels (see the Methods section)^14^. The membrane potential is then defined as *V*_m_ = *V*(*I* = 0). The channel electrical conductances *G*_out_ (outward) and *G*_in_ (inward) are of the order of 1 nS^9^. For the sake of simplicity, the equilibrium potentials are fixed to *E*_in_ = −60 mV (hyperpolarized value) and *E*_out_ = 0 (depolarized value)^32^ in Fig. 2a. These equilibrium potentials depend on the ionic concentrations, which are approximately constant for the short times characteristic of electrophysiological experiments^33^. The number of effective charges involved in the channel gating is *z* = 3 and the threshold potential (midway voltage of activation, i.e. the potential at which the open probability is 0.5) is *V*_th,in_ = *V*_th,out_ = −25 mV in Fig. 2a. These channel characteristics are typical of voltage-gated ion channels^9^,^16^,^34^,^35^.

Figure 2a shows three values of *V*_m_* = V*(*I* = 0) corresponding to the hyperpolarized and depolarized stable values (which are reminiscent of a two-state biological memory) and the central unstable value. Electrical bi-stability is a key characteristic of neural cells^36^ and it is meaningful to consider it in the case of non-neural cells operating over times longer than those characteristic of excitable cells. Moreover, bi-stability has been obtained previously in theoretical^37^,^38^ and experimental studies (lysenin channels inserted into lipid bilayer membranes^39^, hair cell membranes^40^, and skeletal and mouse lumbrical muscle cells^34^).

Figure 2b shows transitions between the hyperpolarized and depolarized membrane potentials induced by modifying the values of the equilibrium potential *E*_in_ (this potential depends on the ionic concentrations^9^). Remarkably, the transition characteristics can be regulated by the conductance ratio *G*_out_/G_in_^14^, which suggests that cells expressing differently the outward and inward rectifying channels characteristics (and then the ratio *G*_out_/*G*_in_) may show different membrane potentials *V*_m_.

Recent reviews on clinical and experimental oncology have considered the concurrent upregulation of sodium channels and downregulation of potassium channels as essential steps to cancer progress via increased sodium inward currents and decreased potassium outward currents (see e.g. Fig. 2 of ref. 7 and references therein). As an illustrative example showing the consequences of specific channel upregulation at the single cell level, consider the physiologically-significant electric potentials *V* < 0 in Fig. 2a. In the present model, upregulation of outward channels and downregulation of inward channels would correspond to the case *G*_out_/*G*_in_ > 1 in Fig. 2b. In this case, the membrane potential *V*_m_ decouples from the normal hyperpolarized value *E*_in_, entering the bi-stability and cell depolarization regime in Fig. 2b. Depolarization is a characteristic of abnormal cells that promotes the initiation of biochemical signal cascades^7^,^27^ which will be ignored in this biophysically-centered approach.

The cell microenvironment characteristics should also be incorporated in the model. We consider here the case of an ion channel blocker because it has been suggested that metastatic activity could be decreased by externally blocking the activity of specific ion selective channels^15^,^28^. This suggestion can be simulated in the model by introducing the blocker concentration *c*(*x*,*y*) in the external cell microenvironment, where *x* and *y* are the spatial coordinates in the cell ensemble (see the Methods section for details). As an illustrative example, the blocking of the outward-rectifying channel can be described by decreasing the channel conductance ratio *G*_out_/*G*_in_ with the blocker concentration. In this way, channel blocking would correspond to the case *G*_out_/*G*_in_ < 1 in Fig. 2b.

### Description of the multicellular electrical states

**Figure 3:**
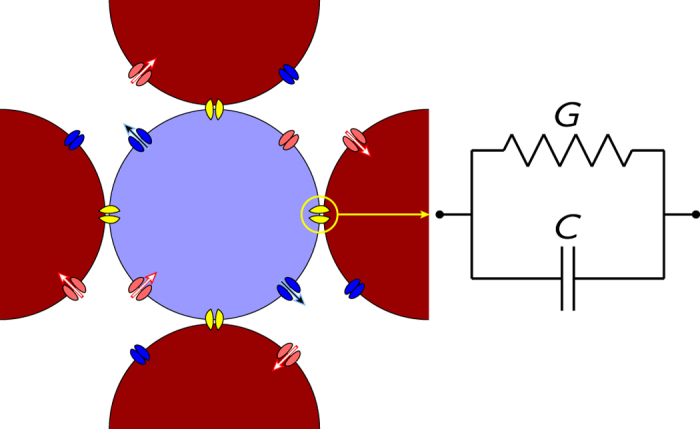
The electrical coupling between cells. Connection of the central cell to the neighboring cells is realized by coupling junctions of effective conductance *G* and capacitance *C*. For typical values of *G* and *C*, the conductance term dominates over the capacitance term^14^.

Intercellular communication has been found defective in abnormal tissues^22^,^23^,^41^,^42^. Hence, in addition to the single cell and microenvironment characteristics, the model should incorporate the effects of intercellular coupling. The long-range electrical coupling in the multicellular ensemble can be simulated by effective conductances (*G*) and capacitances (*C*) arranged in parallel (Fig. 3)^14^. These bioelectrical elements can be realized by the protein channels which act as gap junctions between neighboring cells^13^,^42^,^43^.

The cell ensemble consists of *N* identical model cells simulating a small patch of tissue (we do not consider the problem of tissue growth here). The central cell (i) in Fig. 3 has a time (*t*)-dependent electric potential *V*_i_(*t*) which may have an intermediate value between the low (depolarized) and high (hyperpolarized) membrane potentials in Fig. 2b. The spatial map of electric potentials changes with time because of the local ionic currents established through the junctions of neighboring cells in the multicellular network^14^.

In the multicellular ensemble simulations, we assume that the single cell potential changes only because of the electrical coupling between neighboring cells and ignore local changes in the ionic concentrations (the equilibrium potentials *E*_in_ and *E*_out_ are then constant). This assumption should be valid for the extracellular environment acting as a buffer which fixes the external ionic concentrations^34^. The intracellular ionic concentrations should also be approximately constant in the case of electrical relaxation processes because the number of ions transferred across the membrane to set up typical potential differences is very small compared with the total number of ions in the cell^13^. The above assumptions are equivalent to assuming that the multicellular ensemble dynamics are dictated by the individual cell potentials, which should be valid for short enough experimental times^13^,^14^,^33^,^38^.

Figure 3 shows that the individual mechanisms acting at the cell level (Fig. 2) are coupled together by the electrical network at the multicellular level, which produces the spatial patterns over the cell ensemble. Note that the cells in the ensemble can be at different potentials because of the finite conductance values typical of biological junctions (see Fig. 2)^14^. More details on the dynamic model equations can be found in the Methods section and ref. 14. We will consider now three problems of current interest: the spatial patterns of single cell electric potentials obtained for different degrees of intercellular bioelectric communication, the role of ion channels in the modeling of cancer biophysics, and the spatial distribution of charged nanoparticles over a model tissue^2^,^6^,^7^,^11^,^44^,^45^.

## Results and Discussion

### Normalization of small regions with depolarized cells

**Figure 4:**
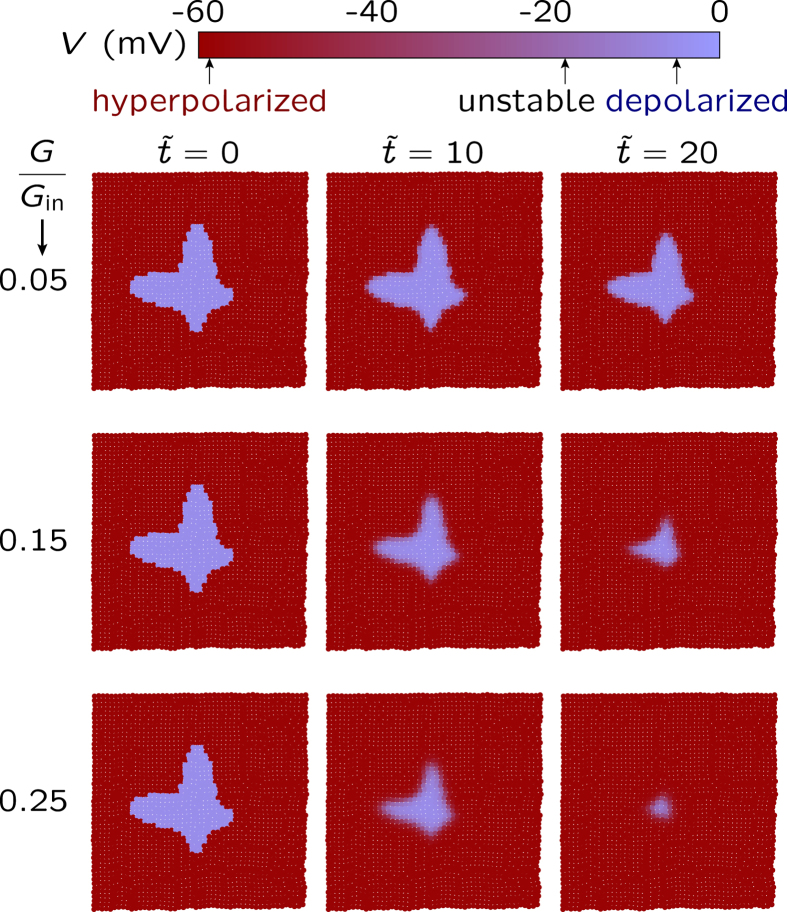
Normalization of small regions with depolarized cells. The hyperpolarization of the spatio-temporal map of cell potentials for the case of a multicellular ensemble with 50 × 50 = 2500 cells and a depolarized central region. The top bar indicates the cell potential values. The individual cell characteristics are those in Fig. 2, with *G*_out_/*G*_in_ = 1.0. The relative gap junction conductance takes values from *G*/*G*_in_ = 0.05 (top) to 0.25 (bottom)^47^. The dimensionless time is defined as 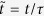, with the characteristic time τ = *C*_0_/*G*_in_ 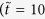 is equivalent to *t* = 1 s for the reference^14^ capacitance *C*_0_ = 100 pF and conductance *G*_in_ = 1 nS). Initially (zero time), the dominant hyperpolarized potential cells (normal state in red) contain a central region of depolarized potential cells (abnormal state in blue). The reversion of this depolarized region to the normal hyperpolarized potential state occurs only when the multicellular coupling due to the junction conductances is strong enough (top panel), emphasizing the importance of the bioelectrical communication between cells in Fig. 3.

The gap junctions in Fig. 3 allow the communication between cells by converting the electrical signals at the single cell level into multicellular states. Figure 4 (bottom panel) shows that the normalization of small regions with abnormal depolarized cells is possible for high enough coupling conductances *G*. However, this normalization process is not achieved for defective intercellular communication (top panel) because the low values of *G* produce cell isolation in this case. The results in Fig. 4 suggest also that a non-uniform distribution of gap junctions may allow the coexistence of spatial regions having increased intercellular communication (high *G* values) with other regions having decreased communication (low *G* values). This question should have clear implications on the long-range gap junctional signaling characteristic of patterning^2^,^6^ and tumorigenesis^46^.

For strong coupling (high *G* values in Fig. 4), the hyperpolarized potential state is forced by the normal cells acting as an electrical buffer for the relatively small number of abnormal cells. As it could be expected, this self-correction of the physiologically corrupted left pattern in Fig. 4 should be difficult when a high number of abnormal cells are present: the local majority rule favors the depolarized, abnormal state in this case^14^.

The low response times obtained suggest that slow changes in the ionic concentrations^34^ should follow the rapid electrical relaxation in Fig. 4. We have not described these diffusional processes which should occur over longer times and larger spatial regions than those characteristic of the rapid electrical relaxation occurring in the small multicellular region in Fig. 4. While the above processes should be incorporated in more complete tissue models, changes in the individual cell potentials can initiate and influence the comparatively slow biochemical pathways which regulate the cell state and proliferation^2^,^6^,^27^.

Experimentally, non-functional junctions and defective intercellular communication are typical of uncontrolled growth regulation in tissues^6^,^23^,^41^. In our model, abnormally low gap junction conductances can isolate depolarized potential cells from the inhibitory electrical signals characteristic of the hyperpolarized potential neighboring cells (Fig. 4, top). Remarkably, the conductance of the gap junctions can be modulated by external agents^13^,^23^,^42^,^43^, which suggests methods to reverse this process. Note finally that isolated cells could eventually proliferate and expand^8^,^23^ but this process would take times much larger than those considered here because of the relatively slow biochemical^13^ and diffusional^34^ processes occurring at the single cell and multicellular levels.

### Effects of channel upregulation

Overexpression and upregulation of specific ion channels in the membrane can alter the normal cell electrical balance, stimulating uncontrolled proliferation^7^,^25^,^28^,^29^. In particular, ion channel upregulation can lead to persistent depolarization of the cell electric potential, modifying the spatial distribution of negatively charged lipids and provoking the clustering of signaling proteins with positive residues around them.^27^ This clustering causes the initiation of biochemical pathways which promote cell proliferation (see Fig. 1 of ref. 27 and references therein).

**Figure 5:**
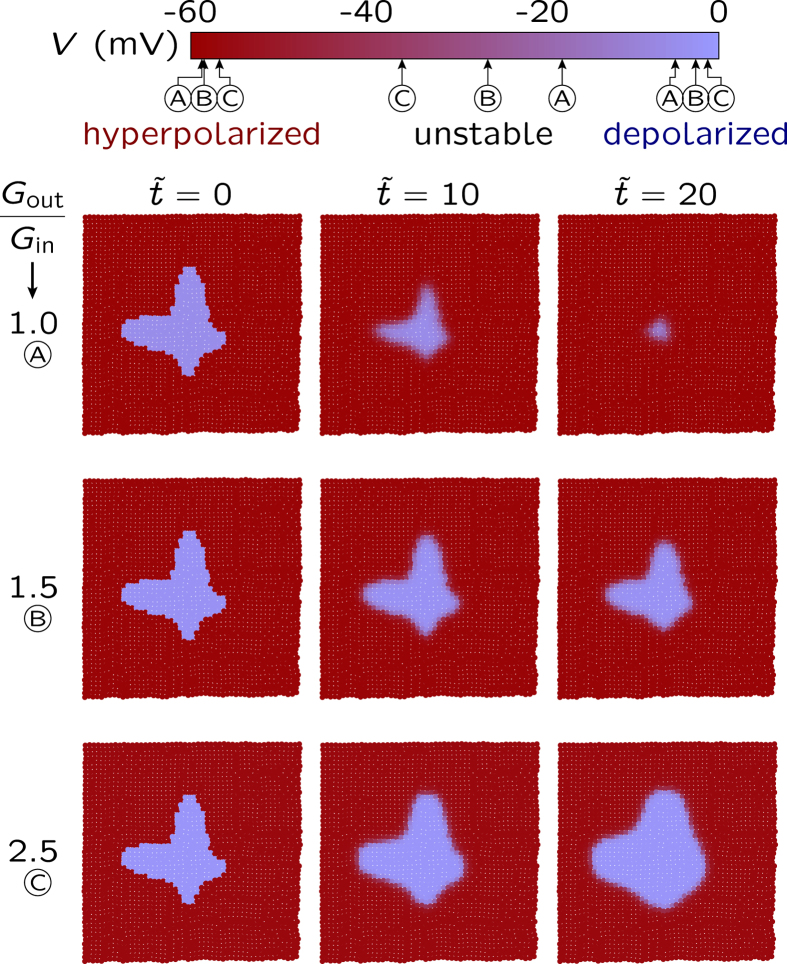
The effect of channel upregulation on normalization. The spatio-temporal map of cell potentials obtained for the cell parameters in Fig. 4 except for the fixed gap junction conductance ratio *G*/*G*_in_ = 0.25 (bottom panel in Fig. 4) and the variable channel conductance ratio *G*_out_/*G*_in_. The increasing values of *G*_out_/*G*_in_ simulate the upregulation of the outward-rectifying (depolarizing) channel while keeping constant the intercellular coupling *G*/*G*_in_. Initially, the dominant hyperpolarized potential cells coexist with a small central region of depolarized cells. The normalization of this central region (top row, *G*_out_/*G*_in_ = 1.0) is no longer possible when the outward-rectifying channel is overexpressed (bottom row, *G*_out_/*G*_in_ = 2.5), which illustrates the importance of the electrical balance between the ion channels. The letters A, B, and C in the cell potential bar (top) indicate the small shifts in the stable membrane potential values which result from the changes in *G*_out_/*G*_in_ (see Fig. 2b).

The upregulation of the outward rectifying channel, simulated here by increasing the conductance *G*_out_ with respect to *G*_in_, promotes depolarization (as shown in Figs 2b and 5). The initially depolarized central region cannot be normalized for high enough values of *G*_out_/*G*_in_. Instead, this abnormally electrical region expands and invades the normal cell region when the outward rectifying channel is up-regulated (bottom row).

Taken together, Figs 2b and 5 indicate that models incorporating specific ion channels with different conductances will show different mono and bi-stability regions^12^,^34^,^38^,^39^,^40^. Figure 5 suggests also that the membrane potential depolarization characteristic of abnormal cells should be possible when certain key channels are up-regulated, in agreement with experiments^27^,^28^,^29^. While these specific channels will depend on the particular biological cell studied, a simple model with only two generic voltage-gated channels qualitatively captures this fact in Fig. 5.

### Blocking of the outward rectifying channels

**Figure 6:**
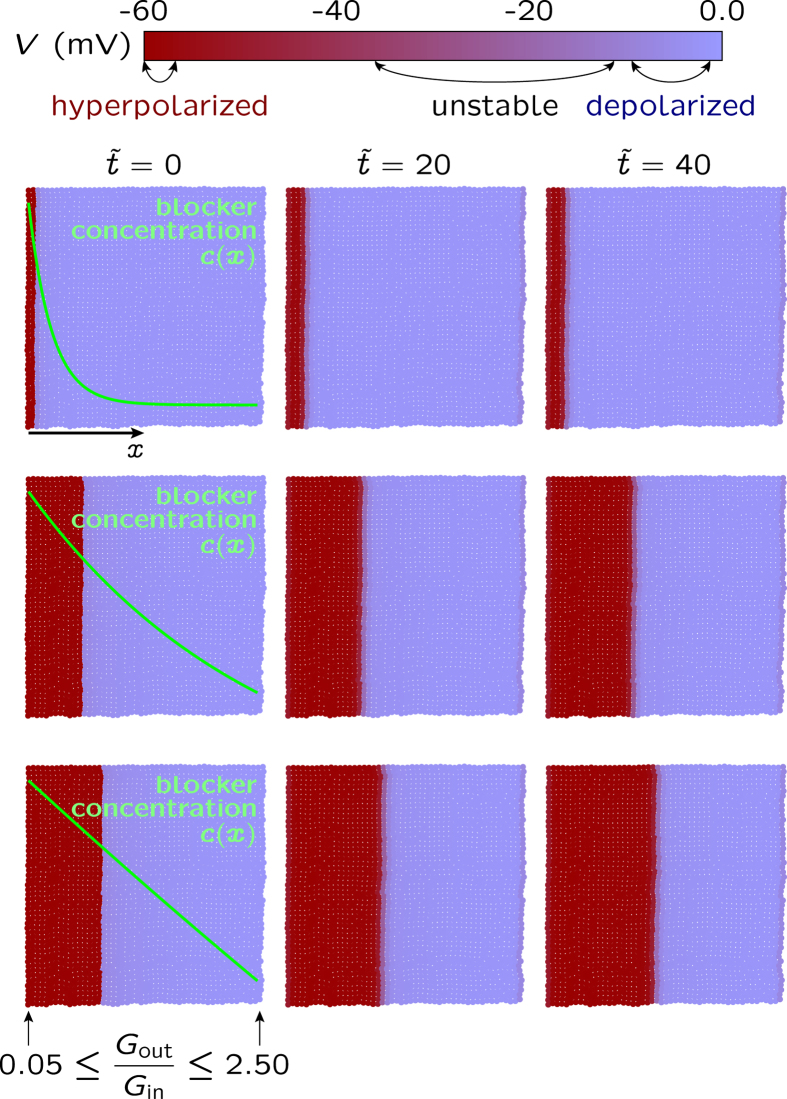
Effects of blocking a specific channel. The spatio-temporal map of cell potentials obtained for the cell parameters in Fig. 5 and the local channel conductance ratio *G*_out_/*G*_in_ regulated by the blocker concentration *c*(*x*), where *x* is the axial coordinate. Three concentration profiles for the blocker of the outward-rectifying (depolarizing) channel are superimposed on the potential maps. The concentration *c*(*x*) produces values of *G*_out_/*G*_in_ which vary between *G*_out_/*G*_in_ = 0.05 (left, maximum blocker concentration) and 2.5 (right, minimum blocker concentration). The axial progression of the hyperpolarized potential region is assisted by the blocker. This result illustrates an external method to suppress the upregulation of the outward-rectifying channel shown in Fig. 5: the normal cell state could be recovered by blocking specific channels. The arrows in the cell potential bar (top) indicate the range of the stable membrane potential values which result from the changes in *G*_out_/*G*_in_ occurring from left to right.

Functional tissues are open systems subject to biochemical, mechanical, and electrical changes in the microenvironment. In Fig. 6, we show that normalization and patterning can be promoted by an external blocking agent whose different concentration profiles *c*(*x*) are superimposed to the map of local potentials. In this case, the blocking of the outward rectifying channel locally decreases the conductance *G*_out_ with respect to *G*_in_, an effect opposite to that shown in Fig. 5 (see the Biophysical Model section for the blocking model and Fig. 2b for the effects of changing the conductance ratio *G*_out_/*G*_in_).

Note that the establishment of the electrical pattern is fast because we do not describe the diffusional relaxation of the blocker, assuming a fully developed steady-state concentration profile *c*(*x*) and a fast channel blocking reaction (the channel responds immediately to the blocker presence). The coupling between cells allows the propagation of the local perturbation caused by the channel blocker at the single cell level to the multicellular ensemble. In this case, the electrical signal propagation through the axial position promotes the gradual transition from the abnormal depolarized state to the normal hyperpolarized potential cell state.

The results suggest that different blockers acting on the conductance of specific ion channels should be able to restore the normal cell polarization by suppressing the upregulation of these channels (compare Fig. 6 with Fig. 5, top row). The extension of the hyperpolarized potential region through the multicellular ensemble should be facilitated by normal, non-defective gap junctions (see Fig. 4). Taken together, Figs 4 and 6 show that the concerted action of an external agent acting at the single cell level and the long-range communication acting at the multicellular level can reduce the extension of the depolarized, electrically abnormal region. Conversely, the blocking of specific channels promoting membrane hyperpolarization rather than depolarization should increase the extension of the depolarized region. It has been observed that cationic nanoparticle-induced blocking of the potassium channels that are responsible for maintaining the membrane potential leads to significant depolarization of CHO and HeLa cells^45^.

We have ignored diffusional effects in Fig. 6. In order to describe the diffusional relaxation of the blocker, we should not use the assumption of a fully developed steady-state concentration profile in Fig. 6. The Methods section presents a simple approach for the coupling of the blocker diffusion equation to the channel blocking reaction. In this approach, the fast electric time in Fig. 4 is replaced by the slow diffusional time τ_d_ = *L*^2^/*D* where *L* is the multicellular ensemble length and *D* is the blocker diffusion coefficient. As it could be expected, time τ_d_ is of the order of hours for typical values of *L* and *D* (see the Methods section) suggesting that the results in Fig. 6 should involve times much longer than those in Fig. 4 when the diffusion processes are included in the model.

### Patterning along predefined spatial directions

**Figure 7:**
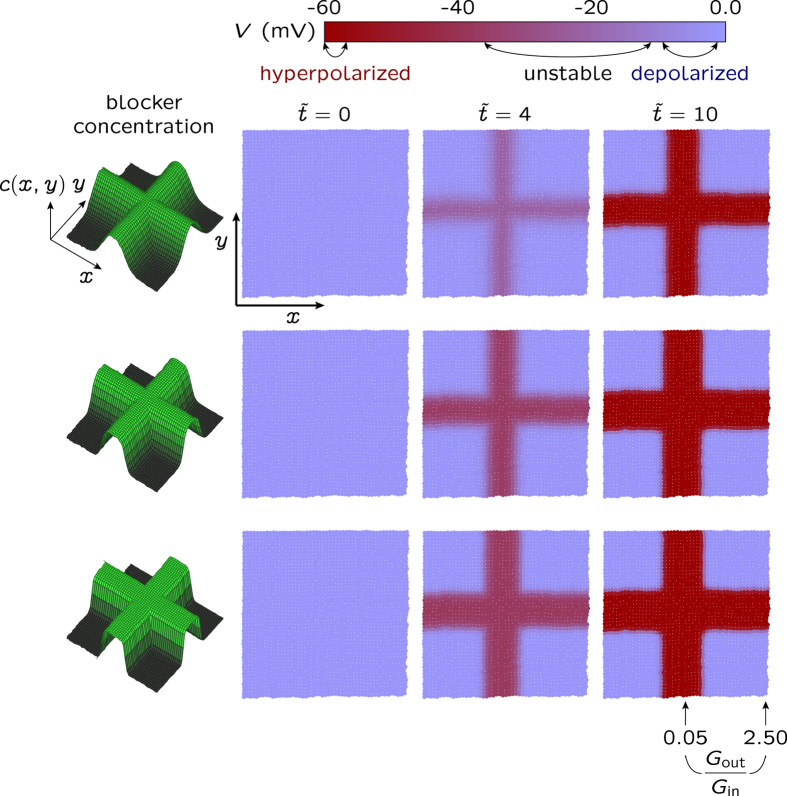
Patterning of the spatio-temporal map of cell potentials caused by a two-dimensional blocker concentration. The map is obtained for the cell parameters in Fig. 6 when the local conductance ratio *G*_out_/*G*_in_ is regulated by the blocker concentration *c*(*x,y*) shown in the left, where *x* and *y* are the spatial coordinates (see the Methods section). The bioelectrical patterning of the multicellular ensemble with hyperpolarized and depolarized potential regions closely follows the blocker concentration *c*(*x*,*y*) acting on the outward-rectifying channel. As in Fig. 6, *c*(*x*,*y*) gives conductance ratios which vary between *G*_out_/*G*_in_ = 0.05 (central cross in the potential map, maximum blocker concentration) and 2.5 (map corners, minimum blocker concentration). The range of membrane potential values obtained from the changes in the above conductance ratio is shown in the cell potential bar (top). This figure illustrates the establishment of electrical multicellular patterns by changing the relative contributions of specific channels to the cell membrane conductance.

The results in Fig. 6 also indicate that the coordinated action of external perturbations acting along predefined spatial directions could produce patterning^14^. Figure 7 addresses this question: the multicellular patterns (right) result now from a two-dimensional spatial profile *c*(*x*,*y*) for the blocking agent (left) in Fig. 6. Note that we assume a fast channel blocking reaction which is locally dictated by the steady-state concentration profile *c*(*x*,*y*): the steepness in the spatial map of electric potential follows closely that of the blocker concentration. Remarkably, the symmetry breaking characteristic of the spatio-temporal patterns shown in Fig. 7 requires no anisotropic electrical couplings^14^ between the neighboring cells in Fig. 3.

Incidentally, it is tempting to speculate that the external blocker can provide a mechanism for establishing a positional information scheme based on bioelectrical signals. All individual cells in the multicellular ensemble shown in Fig. 7 are equivalent but a given cell would be able to read its relative spatial position with respect to the neighboring cells provided that it can sense the locally different electric potentials and fields imposed by the spatial distribution of the blocking agent. In addition to transport processes through the neighboring gap junctions^47^, this electrical sensing could be based on the charged lipids and proteins in the cell membrane which show significant spatial redistributions in response to local electrical fields^9^,^13^,^27^. The multicellular ensemble can then be regarded as a bioelectrically coupled network supported by the intercellular gap junctions allowing the transmission of bioelectrical signals^14^,^46^,^48^. It should be cited here that, in addition to specific morphogens, interactions among cells can be crucial for positional information^48^,^49^.

The present model of slow electrically excitable network incorporates external biochemical signals (blocking agents), individual cell properties (voltage-gated ion channels) and collective coupling mechanisms (gap junctions), giving electrical patterns reminiscent of morphogenetic fields. Remarkably, these mechanisms incorporate not only the spatial distribution of absolute membrane potentials but also the relative differences of these potentials across the gap junctions and their time-dependent changes, as suggested in experimental studies^46^,^48^. We must admit, however, that diffusion-reaction processes with long characteristic times should also be included in the model for quantitative descriptions of patterning^1^,^2^,^21^,^32^.

### Spatial distribution of charged nanoparticles over the multicellular ensemble

**Figure 8:**
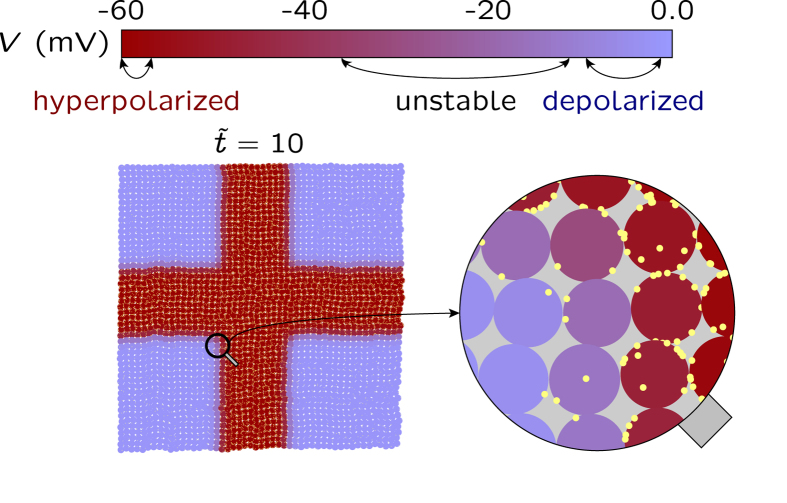
The spatio-temporal map of cell potentials regulates the distribution of charged nanoparticles. A fixed number of positively charged nanoparticles (small circles) are spatially distributed over the map of potentials in Fig. 7 (bottom). The nanoparticles tend to be concentrated around the hyperpolarized potential cells. The inset zooms a small region of the multicellular ensemble composed by 3 × 3 = 9 cells.

The potential use of charged nanoparticles in clinical applications addressed to small tissue regions is receiving much attention^44^,^45^. Figure 8 shows that the map of electric potentials resulting from cell coupling can regulate the distribution of charged nanoparticules over the multicellular ensemble shown in Fig.7 (bottom panel). The number of nanoparticles (small circles) around each individual cell shown in Fig. 8 is scaled to the exponential function of the local electrical potential, assuming a Boltzmann equilibrium for the spatial distribution of particles^9^,^50^. As experimentally observed^44^,^45^, the positive nanoparticles tend to be concentrated around the negatively charged cells. This result indicates that the spatial map of potentials should influence the local uptake of charged nanoparticles over a tissue^44^. Remarkably, the binding of nanoparticles to cells can also disrupt the cell membrane potential^45^ and modify intercellular communication (Fig. 4). These processes suggest external methods to modify the map of potentials alternative to those shown in Figs 5 and 6.

In summary, Figs 4, 5, 6, 7, 8 suggest that the spatial map of electric potentials characteristic of multicellular ensembles can be externally modified by taking advantage of the long-range electrical coupling between cells. The results illustrate electrical normalization caused by multicellular coupling and blocking of specific channels, spatial patterning along predefined directions, and electrically-induced spatial redistribution of charged nanoparticles over small regions of a model tissue. The model considers only two generic voltage-gated channels (ion pumps and active transport, as well as additional ion channels, may be needed for a quantitative description of particular cells^2^,^13^,^34^,^38^). The slow diffusional processes^34^,^51^ which should follow the relatively fast electrical responses obtained here have also been noted (see Fig. 6 and Methods section). Even with these limitations, we have shown the importance of bioelectrical signals, providing results of qualitative value which can be connected with current experimental problems:

1. the spatial map of potentials in Fig. 4 and the single cell local potential domains can be imaged by membrane voltage-reporting dyes (see Figs 8 and 9 of refs 2 and 48). This information should be significant because cancer microenvironment may show long-range bioelectrical signals and gap junctional insulation is involved in tumorigenesis (refs 6, 25 and 42). Also, membrane depolarization may trigger transcriptional changes by regulating morphogens transport^6^;
2. the changes in the single cell membrane potential polarization discussed in Figs 4, 5, 6 should occur when specific channels are up-regulated, down-regulated, or physically blocked (refs 2, 6, 7, 11, 15 and 29, 30, 31). In principle, decreasing the activity of a particular channel will modify the membrane polarization state it promotes, as shown in Fig. 6. However, addressing only a specific channel may be misleading because the membrane polarization is a physiological characteristic that results from the non-linear combination of different ion transporters which operate in a changing microenvironment^6^,^9^ and suppressing a target channel may be compensated by other redundant channels^48^. In our simple model, this compensation could be achieved in different ways: modifying the gap junction conductance (Fig. 4), shifting the balance between the outward- and inward-rectifying channels functionalities (Fig. 5), and blocking specific channels (Fig. 6). Increasing the complexity of the model with other ion channels and pumps should lead to additional feedback mechanisms. Persistent changes in the membrane polarization may then require acting simultaneously over different transporters because bioelectrical phenomena occur at a different level than cell genetics^48^;
3. the role of ion channels in the modeling of cancer biophysics has recently been noted^10^,^11^ and ion channel blockers relevant to the effects shown in Fig. 6 are available (see e.g. refs 15 and 45). The blocking channel approach should provide key information on basic cellular processes. Because of the broad functionality of ion transporters in living systems^9^, however, this approach can promote also serious side effects (e. g. cardiac arrhythmias^15^) and is limited to the channel conducting mechanism only^31^,^52^.
4. the electrical characteristics associated with the spatial distribution of external morphogens over the multicellular ensemble in Fig. 7 can be of significance for patterning (see refs 2 and 32 and references therein). In particular, the results indicate that in addition to specific morphogens, interactions among cells can be crucial for positional information, as noted in experimental studies^48^,^49^;
5. the map of electric potentials resulting from cell coupling in Fig. 8 locally regulates the experimental uptake of charged nanoparticles over tissues (see Fig. 2 of ref. 44 and 1 of ref. 45). These nanoparticles can disrupt the cell membrane potential^45^ and modify intercellular communication (see Fig. 6 for the possible effects of nanoparticle blocking of specific channels).

## Conclusions

Most theoretical descriptions of the spatial patterns characteristic of positional information and cancer processes are single-cell centered and emphasize biochemical signals and pathways. While these approaches have been useful, ionic species are crucial for cell function and then questions concerning bioelectrical signals, ion channels, cell membrane potentials, and intercellular electrical communication naturally arise^2^,^4^,^8^,^14^,^18^,^24^. Collective multicellular states may show electrical coupling mechanisms that are not readily deduced from biochemical descriptions at the individual cell level^8^,^14^,^22^,^26^.

We have explored the consequences of a simple biophysical model for electrically-connected cells based on a reduced number of concepts: the cell membrane potential (obtained from two generic voltage-gated ion channels favoring cell hyperpolarization and depolarization), the multicellular electrical coupling (supported by a network of gap junction channels), and the biochemical coupling of the cell ensemble with the external microenvironment (realized by a channel blocker). Specific biochemical signals and pathways characteristic of real biological problems are ignored but new biophysical insights amenable to experimental analysis are obtained on the basis of the building blocks characteristic of the multicellular electrical circuitry: the protein ion channels and coupling junctions of the cell membrane.

The multicellular electrical patterns are spatio-temporal maps of single cell potentials. These maps are of significance for positional information processes based on the coupling of the individual potentials with the spatial distribution of a blocker^1^,^53^. Also, the electrical patterns show the importance of ion channels in the modeling of defective intercellular communication and cancer biophysics^10^,^25^,^27^. Knowledge of the local map of potentials should facilitate procedures to collectively change the bioelectrical characteristics of small tissue regions, e.g. by the external modulation of the single cell states by blockers of specific ion channels^9^,^15^,^28^,^54^. The spatial distribution of charged nanoparticles over tissues, a subject of current clinical interest, is also regulated by the electric potential map: the membrane potential modulates the binding of the nanoparticles to the cell surface^44^. All in all, the present simulations are based on a reduced number of concepts and show significant connections with current biophysical problems.

## Methods

### Model Simulations

The inward-rectifying channels allow large inward currents at potentials more negative than the equilibrium potential 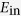 (low outward currents are obtained at potentials less negative than 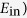
9,14. The phenomenological *I*–*V* curve is


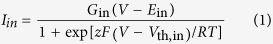


where *R, F*, and *T* are the gas constant, the Faraday constant, and the temperature, respectively. *V*_th,in_ is the threshold potential (the potential at which the average relative conductance of the channels is 0.5)*, G*_in_ is the maximum channel conductance, and *z* is the effective number of charges for gating^9^,^14^. The dimensionless current 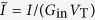 allows to write the above equation as


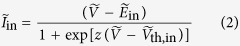


where the dimensionless potentials are defined as 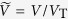, 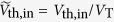, and 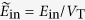. The thermal potential *V*_T_ = *RT*/*F* = 27 mV (*T* = 310 K). Typical dimensionless values are 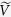 = 2 for *V* = 54 mV and 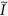 = 2 for *I* = 54 pA, obtained with a channel conductance *G*_in_ = 1 nS^14^. The *I*–*V* curve of the outward-rectifying channel is similar to that of Eq. 2, except for the changes 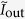 for 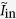, 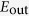 for 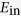, *V*_th,out_ for *V*_th,in_, and −*z* for *z*. Finally, the cell membrane potential *V*_m_ results from the zero current equation^14^:


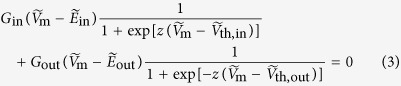


The potential 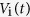 of cell i changes with time according to (see Fig. 3):


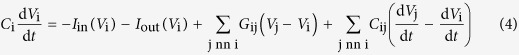


where 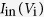 is given by Eq. 1. The cell capacitance is 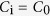 (constant; see Fig. 4). The conductance and capacitance of the gap junction which couples cells i and j in Fig. 3 are 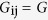 and 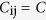, respectively (see Fig. 4). The summations are restricted to the cell nearest neighbors (nn) in Fig. 3. The reference potential in the extracellular microenvironment is assumed to be zero. Note that the local potentials 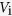 are regulated by the single cell inward- and outward-rectifying ion channels (the first two terms of Eq. 4) and the cell coupling with the other cell potentials in the multicellular ensemble (the last two terms of Eq. 4). Using dimensionless variables, the above equation is


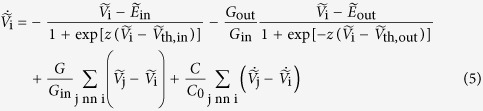


The dot indicates derivation with respect to the dimensionless time 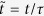, where the characteristic time τ = *C*_0_/*G*_in_ (see Fig. 4). In most cases, the coupling conductance term dominates over the capacitance term for typical values of *G* and *C*^14^ and is then omitted in the simulations.

The blocking of the outward-rectifying channel is simulated by changing locally the conductance ratio function [*G*_out_/*G*_in_](*x*) with the axial position *x* according with the blocking concentration functions *c*(*x*) shown in Fig. 6. In each case, the functional parameters are chosen to give [*G*_out_/*G*_in_](*x* = 0) = 0.05 (left position in the multicellular ensemble) and [*G*_out_/*G*_in_](*x* = *L*) = 2.50 (right position in the multicellular ensemble of length *L*). In the case of Fig. 7, *G*_out_*/G*_in_ is changed according to exponential functions of the spatial coordinates *x* and *y* whose parameters are chosen to give the minimum and maximum values shown in this figure.

The potentials 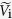 in Eq. 5 are to be solved for the cells 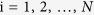 in order to obtain the spatial map of cell potentials as a function of time. The numerical solution of Eq. 5 for potential 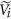 can be obtained using matrix algebra in the form:


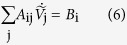


where


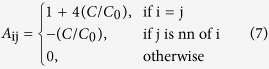


and


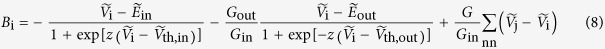


 The numerical algorithm proceeds as follows:

1. Calculate matrix *A*_ij_, which remains constant throughout the calculation.
2. Fix a constant time step 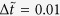.
3. Calculate the cell potential of the isolated cells assuming a constant equilibrium potential and the parameters introduced in Fig. 2.
4. The initial values 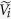 are set to either the hyperpolarized or the depolarized potential, which correspond to the stable potentials of the cells.
5. The numerical calculations proceed until a prescribed final time is reached, with the change 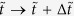 for each time step, following the scheme:

- Calculate *B*_i_
- Solve the system 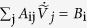 to obtain 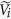
- Update the value of 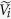 as 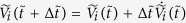.

Equations (1)–(8) do not include diffusional terms. The diffusion of the blocker and the local equilibrium reaction causing the blocking of the outward-rectifying channel can be included in the model using the equations:


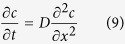


and


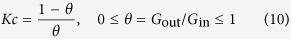


where *D* is the blocker diffusion coefficient and *K* is the blocking constant. An additional equation for the mass conservation should be added if the blocker availability is limited. Note that the blocker concentration *c*(*x*,*t*) and the conductance ratio in Eq. (10) are now functions of the position *x* and time *t*. Equation (10) assumes that the blocking process forces the conductance *G*_out_ to take values between zero (maximum blocking) and *G*_in_ (minimum blocking). Introducing dimensionless variables in Eq. (9) leads to the characteristic diffusional time τ_d_ = *L*^2^/*D*. For an ensemble length of 100 cell diameters, *L* is of the order of 10^−3^ m. Assuming a typical diffusion coefficient of the order of *D* = 10^−10^ m^2^/s, we obtain τ_d_ = 10^4^ s which is about 3 h. As expected, diffusion times should be much larger than electrical times for the multicellular ensemble.

## Additional Information

**How to cite this article**: Cervera, J. *et al*. Bioelectrical Signals and Ion Channels in the Modeling of Multicellular Patterns and Cancer Biophysics. *Sci. Rep.* **6**, 20403; doi: 10.1038/srep20403 (2016).
